# The medico-legal interpretation of diatom findings for the diagnosis of fatal drowning: a systematic review

**DOI:** 10.1007/s00414-024-03397-8

**Published:** 2025-01-14

**Authors:** Alexander Tyr, Philippe Lunetta, Brita Zilg, Carl Winskog, Nina Heldring

**Affiliations:** 1 Swedish National Board of Forensic Medicine, Retzius v. 5, 171 65 Stockholm, Stockholm, 171 65 Sweden; 2 Department of Biomedicine, Forensic Medicine, University of Turku, Kiinamyllynkatu 10, Turku, 20520 Finland; 3 Department of Oncology-Pathology, Karolinska Institutet, Retzius v. 3, Stockholm, 171 77 Sweden; 4 Swedish National Board of Forensic Medicine, Medicinaregatan 18 C, 413 90 Göteborg, Sweden

**Keywords:** Autopsy, Forensic medicine, Standardization, Taxonomic classification, Post-mortem submersion, Positive controls, Negative controls, Lungs

## Abstract

The diagnostic use of the diatom test for drowning has been under investigation for more than a century. Despite continuing research, its true usefulness remains controversial and under debate. Data regarding the extent to which diatoms can penetrate the lungs and other organs of drowning victims are conflicting; similar discrepancies exist as to the presence of diatoms in the organs of living individuals; and as to the occurrence of postmortem (PM) contamination. To shed light on current understanding, we conducted a systematic review based on the Preferred Reporting Items for Systematic Review and Meta-Analysis (PRISMA) to investigate how the presence of diatoms PM may be interpreted during medico-legal investigations of drowning. Following sequential screening of records based on our predetermined eligibility criteria, we assessed scientific evidence and risk of bias by use of the SPICOT framework. A total of 17 studies reporting diatom concentrations in victims of drowning, in non-drowned controls, and in non-drowned immersed controls were eligible for this review. Our findings suggest that diatom testing may be of use in medico-legal investigations, although its evidentiary value remains uncertain because both quantitative and qualitative results from the literature are insufficiently comparable. Variations in study design, methodology and reporting approach also fail to provide a comprehensive understanding of the significance of false-positive and false-negative results. Further research is warranted on antemortem and PM contamination, and on standardized autopsy and laboratory procedures, as well as on automated and certified diatom-counting and -identification systems. Moreover, since diatom taxonomy lies outside the specialty of forensic medicine, we underscore that collaboration with expert diatomologists is necessary for analysis and interpretation. Until these issues are adequately addressed, the evidentiary value of diatom testing for the diagnosis of drowning will continue to remain elusive and contentious.

**Supplementary Information:**

The online version contains supplementary material available at 10.1007/s00414-024-03397-8.

## Introduction

Diagnosing drowning as the cause of death (COD) requires a comprehensive assessment of circumstantial evidence, pathological findings, and the victim’s medical history [1]. However, autopsy, histological, and biochemical analyses often lack specificity and exhibit significant variability in suspected drowning cases [2, 3]. Given these challenges, diatom analysis emerged over a century ago as a potential supportive tool in medico-legal investigations of drownings [4, 5] yet, its diagnostic value remains debated.

Diatoms are ubiquitous microscopic single-celled or colonial algae ranging from less than 20 μm to more than 500 μm in size, and are composed of two interconnected siliceous units, known as valves [6]. Although these organisms have evolved to thrive in all types of aquatic environments, with over 50,000 identified species residing either suspended in water columns (planktonic) or on ocean floors and river beds (benthic), they also occur in the air and soil [7].

Diatom testing PM assumes that, upon drowning, a living person will inhale waterborne diatoms that penetrate the lungs that cross the alveolar-capillary barrier at ruptured sites. The diatoms are then carried via the bloodstream to “closed organs” such as the brain, liver, kidneys, spleen, and bone marrow [8, 9]. However, the diatom method has attracted criticisms that have plagued its acceptance within the field [10, 11]. First, that lungs and closed organs of drowned victims are sometimes devoid of diatoms. Secondly, that diatoms may also be detected in the lungs and/or closed organs of victims, found either on dry land or immersed in water (PM), who have died from causes other than drowning [5, 12–14].

Such contradictory findings underscore the complexity of diatom analysis and the ongoing controversy regarding its reliability and robustness. Use of diatom testing is therefore infrequent in medico-legal investigations of fatal drowning and is restricted to a limited number of countries and institutions, with many experts criticizing its reliability. However, with the advancement of methodological rigor and growing emphasis on evidence-based practice in forensic medicine during recent decades, a systematic and collective analysis of published results is warranted.

In this systematic review of the English language literature, we examine diatom concentrations in drowned, non-drowned, and non-drowned immersed victims, taking into consideration procedures involving sampling, extraction, and analysis. We also consider potential effects of antemortem (AM) and PM diatom contamination. The aim of the present study is to understand the collective evidence of how diatom findings are interpreted during PM medico-legal investigations of fatal drowning, in order to guide further studies, and to develop recommendations on best practice for diatom testing.

## Methodology

### Research question

Our systematic literature review design was based on the Preferred Reporting Items for Systematic Review and Meta-Analysis (PRISMA) [15, 16] and aims to address the question: “how may the presence of diatoms PM be interpreted to serve medico-legal investigations of fatal drowning?

### Search strategy and data sources

Search terms were determined by the research question. Following identification of relevant search terms, query strings were constructed using the Boolean operators “AND” and “OR” (Table 1). Records were collected from the databases of PubMed, CINAHL, and Web of Science, from their inception until 30 May 2024 to maximize search yield. Further items were also identified from the reference lists of selected records if deemed relevant.

**Table 1:** Search strategy

**Set**	**Intervention**	**PubMed**	**CINAHL**	**Web of Science**
**1**	**Forensic autopsy**	"Autopsy"[Title/Abstract] OR "autopsy findings"[Title/Abstract] OR "post-mortem"[Title/Abstract] OR "necropsy"[Title/Abstract] OR "postmortem examination"[Title/Abstract] OR "post-mortem changes"[Title/Abstract] OR "Autopsy"[MeSH Terms] OR "Postmortem Changes"[MeSH Terms] OR "forensic science"[tiab] OR "forensic pathology"[tiab] OR "medicolegal investigation"[tiab] OR "medico-legal"[tiab] OR "judicial"[tiab] OR "forensic medicine"[tiab] OR "forensic autopsy"[tiab] OR "forensic investigation"[tiab] OR "death investigation"[tiab] OR "forensic nursing"[tiab] OR "forensic examination"[tiab] OR "forensic analysis"[tiab] OR "forensic report"[tiab] OR "forensic findings"[tiab] OR "forensic evidence"[tiab] OR "forensic sciences"[Mesh] OR "jurisprudence"[Mesh]	(TI "Autopsy" OR AB "Autopsy findings" OR TI "post-mortem" OR AB "post-mortem" OR TI "necropsy" OR AB "necropsy" OR TI "postmortem examination" OR AB "postmortem examination" OR TI "post-mortem changes" OR AB "post-mortem changes" OR TI "forensic science" OR AB "forensic science" OR TI "forensic pathology" OR AB "forensic pathology" OR TI "medicolegal investigation" OR AB "medicolegal investigation" OR TI "medico-legal" OR AB "medico-legal" OR TI "judicial" OR AB "judicial" OR TI "forensic medicine" OR AB "forensic medicine" OR TI "forensic autopsy" OR AB "forensic autopsy" OR TI "forensic investigation" OR AB "forensic investigation" OR TI "death investigation" OR AB "death investigation" OR TI "forensic nursing" OR AB "forensic nursing" OR TI "forensic examination" OR AB "forensic examination" OR TI "forensic analysis" OR AB "forensic analysis" OR TI "forensic report" OR AB "forensic report" OR TI "forensic findings" OR AB "forensic findings" OR TI "forensic evidence" OR AB "forensic evidence"	("Autopsy" OR "autopsy findings" OR "post-mortem" OR "necropsy" OR "postmortem examination" OR "post-mortem changes" OR "forensic science" OR "forensic pathology" OR "medicolegal investigation" OR "medico-legal" OR "judicial" OR "forensic medicine" OR "forensic autopsy" OR "forensic investigation" OR "death investigation" OR "forensic nursing" OR "forensic examination" OR "forensic analysis" OR "forensic report" OR "forensic findings" OR "forensic evidence" OR "forensic sciences" OR "jurisprudence")
	**Hits**	**437,747**	**12,179**	**220,104**
**2**	**Drowning**	"drowning"[tiab] OR "near-drowning"[tiab] OR "immersion"[tiab] OR "water inhalation"[tiab] OR "submersion"[tiab] OR "drowned"[tiab] OR "wet drowning"[tiab] OR "dry drowning"[tiab] OR "secondary drowning"[tiab] OR "immersion syndrome"[tiab] OR "immersion pulmonary edema"[tiab] OR "drowning victim"[tiab] OR "drowning death"[tiab] OR "Drowning"[Mesh]	TI "drowning" OR AB "drowning" OR TI "near-drowning" OR AB "near-drowning" OR TI "immersion" OR AB "immersion" OR TI "water inhalation" OR AB "water inhalation" OR TI "submersion" OR AB "submersion" OR TI "drowned" OR AB "drowned" OR TI "wet drowning" OR AB "wet drowning" OR TI "dry drowning" OR AB "dry drowning" OR TI "secondary drowning" OR AB "secondary drowning" OR TI "immersion syndrome" OR AB "immersion syndrome" OR TI "immersion pulmonary edema" OR AB "immersion pulmonary edema" OR TI "drowning victim" OR AB "drowning victim" OR TI "drowning death" OR AB "drowning death"	("drowning" OR "near-drowning" OR "immersion" OR "water inhalation" OR "submersion" OR "drowned" OR "wet drowning" OR "dry drowning" OR "secondary drowning" OR "immersion syndrome" OR "immersion pulmonary edema" OR "drowning victim" OR "drowning death")
	**Hits**	**38,068**	**6,215**	**95,401**
**3**	**Diatoms**	"diatoms"[tiab] OR "diatom test"[tiab] OR "diatom analysis"[tiab] OR "diatom identification"[tiab] OR "diatom detection"[tiab] OR "diatom testing"[tiab] OR "Diatoms"[Mesh]	TI "diatoms" OR AB "diatoms" OR TI "diatom test" OR AB "diatom test" OR TI "diatom analysis" OR AB "diatom analysis" OR TI "diatom identification" OR AB "diatom identification" OR TI "diatom detection" OR AB "diatom detection" OR TI "diatom testing" OR AB "diatom testing"	("diatoms" OR "diatom test" OR "diatom analysis" OR "diatom identification" OR "diatom detection" OR "diatom testing")
	**Hits**	**8,701**	**71**	**29,145**
**4**	**#1 AND #2 AND #3**	**198**	**17**	**157**

*Tiab* = Title/Abstract

*MeSH* = Medical Subject Headings

*TI* = Title

*AB* = Abstract

### Eligibility criteria

The database search was limited to English-language original studies published in peer-reviewed journals. Eligibility criteria were based on the population, intervention, comparison, and outcome (PICO) determined by our research question. Studies needed to include deceased individuals undergoing autopsy and for whom a diatom test was performed to determine the diatom concentrations in organ tissues. Individuals succumbing to delayed drowning in hospital were excluded, where possible. We evaluated studies with and without comparator populations for their risk of bias to determine their inclusion. Studies only providing a binary diatom analysis (yes/no), or in which no concentration could be determined were excluded. A diagnosis (or exclusion) of drowning as a COD needed to be based on circumstantial evidence and on PM findings other than the diatom test to avoid circular reasoning, where possible. Records also needed to include a discussion regarding diatom findings, and their interpretation for the diagnosis of drowning as COD.

### Selection of evidence

The data was exported into Microsoft Excel (Office 2019) for removal of duplicates and further selection. Articles were systematically screened for relevance, first by title, then abstract, and finally in full-text by two authors of this study independently (A.T., N.H.). Any disagreements were resolved during consensus discussions. Only original articles were selected. Editorials, commentaries, case reports, technical protocols, conferences proceedings and epidemiological studies were excluded.

### Study evaluation

To ensure that only records fulfilling established scientific criteria were selected, studies were evaluated independently using the SPICOT framework [17] by two authors of this study (A.T., N.H.). SPICOT evaluates the scientific evidence and risk of bias in the forensic literature by assessing the **s**tudy design, study **p**opulation, **i**ntervention, **c**ontrol/comparison, **o**utcome, and **t**ime aspect.

All studies were evaluated in each of those categories to determine a combined level of evidence and risk of bias categorized as described in Tyr et al. [17]. If variations in scores impacted SPICOT classification, consensus discussions were held to decide final score by the two authors (A.T., N.H.). Studies that both researchers identified as having scored SPICOT-low (0–9 points) were excluded.

As to the risk of bias assessment, we examined in each study whether the study population had first been defined and its sample size (*n*) specified and which tissues had been sampled (lungs, closed organs). The various comparisons and control methods assessed, included whether the study had compared drowning subjects with non-drowned individuals found on land and/or in water, whether water samples from the putative drowning site were analyzed, and if a positive diatom control test (spiking test, to address potential loss of diatoms during sample preparation), and a negative diatom control test (to address potential contamination issues) had been conducted during laboratory procedures to examine potential methodological shortfalls. The sample size of the control groups (*n*) was also examined. We also evaluated whether tissue sampling at autopsy, tissue digestion and centrifugation procedures, and microscopy analysis methods had been defined, and whether such procedures had been compared in single studies. Finally, we identified whether taxonomic analysis of diatoms found in PM tissues and taxonomic comparisons with diatom found in the putative drowning media had been performed and if so, by an expert diatomologist or not.

### Data extraction

A summary of information extracted from the selected studies is detailed in Table 2. This includes publication type and date with the study design. We extracted details surrounding study population and controls, including their size and COD. In addition to tissue sampling at autopsy, we also retrieved details on laboratory procedures (tissue digestion, centrifugation, and washing steps), and on diatom examination under microscopy, alongside information relating to positive (spiking) and negative controls. Quantification of diatom content in samples was recorded and extrapolated, where possible, as diatom concentrations (number of diatoms/10 g of tissue). We also considered narratives regarding the interpretation of results, in addition to analysis and reporting approaches.

**Table 2:** Summary of information extracted from studies

Domain	Criterion
Publication type	Original articles
Publication reliability	Peer-reviewed publication
	Date of publication
Data sources	Human model, postmortem
Study design	Descriptive, correlation, causal-effect or experimental
Population/sample study	Representativeness of the population/sample
	Tissue samples taken during autopsy
	Size of population/sample
Intervention	Diatom test procedure/method
	Tissue size (g) analysed
Controls	Drowning (drowning, isolation, medium)
	Laboratory (diatom digestion, contamination)
	Size of control group(s)
	Aliquot procedure
Assessment	Analysis method
	Reporting system
Outcome	Description of the outcome
	Quantification of diatom concentrations
	Interpretation of results

### Ethical considerations

No studies with living human participants or animals were performed by the authors for the purpose of this study.

## Results

### Study selection

**Fig. 1:**
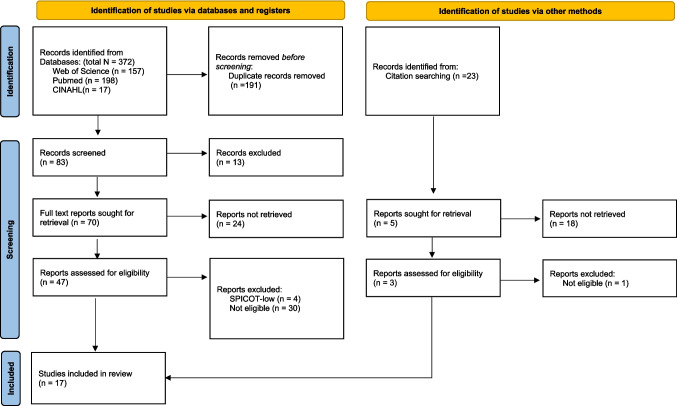
Flow chart detailing study selection

Search results revealed a total of 372 records, of which 198 were from PubMed, 157 from Web of Science, and 17 from CINAHL. Twenty-three additional records were identified from searching the citations of the selected articles. Following removal of duplicates (191), and screening of titles and abstracts for relevance, we retrieved a total of 75 full-text articles to be assessed for eligibility. From these, we deemed 30 studies not eligible, and four were excluded due to being considered SPICOT-low [12, 13, 18, 19]. In total, 17 original studies, all published between 1994 and 2023, were eligible for this review. The selection process is detailed in Fig. 1 according to PRISMA guidelines.

### Risk of bias assessment

The risk of bias assessment is represented in Table 3. All the selected studies had defined study populations, with 13 (76%) collecting diatom samples from multiple organs such as lungs and closed organs. In eight (47%) of the selected studies, the population size exceeded 20 individuals.

**Table 3:** Risk of bias of selected studies

Reference	Population			Control/comparison					Assessment	
	Defined^1^	Tissue selection^2^	Quantity^3^	Control for drowning^4^	Drowning medium comparison^5^	Positive control^6^	Contamination control^7^	Quantity^3^	Methods^8^	Taxonomic classification^9^
Ludes et al. (1994)	+	+ + +	+	+	+		+	+	+ +	+ +
Kristic et al. (2002)	+	+ + +	+ +	+				+	+	
Takeichi and Kitamura (2009)	+	+	+						+	+
Ago et al. (2011)	+	+ + +	+	+ +	+			+	+	+
Bartolotti et al. (2011)	+	+ + +	+ +	+				+ +	+	
Kakizaki et al. (2011)	+	+ + +	+ +	+ + +	+		+	+	+	+
Lunetta et al. (2013)	+	+ + +	+	+ +	+		+		+	+
Lin et al. (2014)	+	+	+	+	+		+	+	+	+
Kakizaki and Yukawa (2015)	+	+ + +	+				+		+ +	+
Fucci et al. (2015)	+	+ + +	+						+ +	+
Zhao et al. (2016)	+	+	+ +	+ +	+		+	+	+	
Zhao et al. (2017)	+	+ + +	+ +	+ +	+		+	+	+	
Shen et al. (2019)	+	+ + +	+ +	+ +				+ +	+	+
Kihara et al. (2021)	+	+	+ +	+ +	+			+	+	
Kakizaki et al. (2022)	+	+ + +	+		+		+		+	+
Sonoda et al. (2022)	+	+ + +	+ +	+ + +	+			+	+	+
Hagen et al. (2023)	+	+	+			*	+		+	

^1^Defined (+)

^2^Tissue selection, lungs only (+); single closed organs (+ +); multiple organs (+ + +)

^3^Sample size: < 20 (+); and > 20 (+ +)

^4^Non-drowning cause of death comparison on land (+); immersed (+ +); or both (+ + +)

^5^Diatom analysis performed in drowning medium for comparison (+)

^6^Postive control for diatom concentrations performed during isolation (+)

^7^Control for contamination performed during isolation (+)

^8^Assessment procedure defined (+); by multiple assessment methods (+ +)

^9^Taxonomic classification performed (+); by diatomologist (+ +)

*Positive control examining evaporation of diatoms during digestion

Although 12 (71%) of the 17 studies included at least one control group to compare with the putative drowning cases, five (29%) studies lacked any control to be tested against drowning as COD. Among the 12 studies with controls, four included control subjects who died from causes other than drowning and were found on dry land, whereas six included subjects who died from causes other than drowning but who were exposed to PM immersion/submersion (Table 3). Only two studies investigated all three study groups (drowned, non-drowned on land, and non-drowned immersed/submerged). One study considered only cases with the non-drowned on dry land vs. non-drowned immersion/submersion. Of the 12 studies, only two examined populations larger than 20.

Of the 17 selected studies, only nine (53%) compared diatom findings from tissue samples with those found in the putative drowning medium (Table 3). In total, nine (53%) studies considered a negative control for contamination during laboratory procedures, but none conducted a positive control (diatom spike) to assess loss of diatoms during tissue digestion and centrifugation. However, one study did mention the use of a positive control to examine evaporation of diatoms during digestion. Overall, 14 studies (82%) used a single isolation method, consisting of either an acid, enzymatic, or microwave digestion process. The latter was used in conjunction with vacuum filtration and automated scanning electron microscopy (MD-VF-SEM); the remaining three studies compared two of these methods against each other. Although 11 (65%) studies conducted a taxonomic classification, only one reported that classification was performed by a trained/certified diatomologist (Table 3).

### Characteristics of individual studies

Characteristics of the main individual sources of evidence are detailed in Table 4. In terms of study design, of the 17 selected studies, 13 (76%) exhibited a correlation design, with three (18%) considering a causal effect, and the remaining one being a retrospective descriptive study. Eastern Asia was the geographic location of occurrence for 11 studies and the remaining six (35%) locations were spread over Europe, grouped into two studies from central Europe, two southern studies, one eastern study and one northern Europe study (Table 4). Of the 17 studies, type of drowning media (salt content) was specified in 14 (82%), seven focused on both salt- and fresh-water drownings, four on tap-water drownings, two on freshwater drownings, two on brackish-water drownings, and one on saltwater drownings. Only one study used a diatom-enriched medium.

**Table 4:** Characteristics of studies

Reference	Total study population (*n*)	Aim of study	Study design	Drowning medium		Volume (g) of samples taken at autopsy*						Laboratory procedures		Analysis and reporting metrics	
				Geographical region	Medium type	Lungs	Liver	Spleen	Kidney	Brain	Bone marrow	Digestion method(s)	Transferred aliquot for analysis	Analysis method	Diatom units counted/reported
Ludes et al. (1994)	17	To compare quantitative and qualitative diatom analysis of water samples from drowning sites and post-mortem tissue samples	Correlation	Continental Europe	SW and FW	10	10	-	10	10	-	AD (HNO₃) and ED (Proteinase K)	All sediment (100 µl/slide)	LM	Whole diatoms and fragmented frustules
Kristic et al. (2002)	23	To analyze possible entry pathways into tissues using an experimental model of drowning and comparing to human drownings	Correlation	Eastern Europe	SW	2	2	-	2	2	2	AD (H_2_O_2_ + H_2_SO_4_)	Supernatant	LM	-
Takeichi and Kitamura (2009)	6	To examine the application of enzymatic digestion for diatom detection in formalin-fixed organs obtained at autopsy	Correlation	Eastern Asia	-	10	-	-	-	-	-	ED (Proteinase K)	Sediment	LM	-
Ago et al. (2011)	10	To explore the utility of the diatom test as a supplementary diagnostic tool for bathwater drowning	Descriptive	Eastern Asia	TW	20	20	20	20	-	-	AD (H_2_O_2_ + H_2_SO_4_)	All acid-free sediment	LM	Whole diatoms and fragments of frustules
Bartolotti et al. (2011)	65	To investigate the presence of diatoms in the tissues of subjects who died from causes other than drowning and compare them with those recovered from water	Correlation	Southern Europe	SW and FW	5	-	-	-	-	10	AD (HNO₃)	Entire pellet (300 µl/slide)	LM	Whole diatoms and fragments of frustules
Kakizaki et al. (2011)	24	To examine the presence of freshwater bacterioplankton in the blood samples of immersed and non-immersed cadavers and correlate their presence with the type of water aspirated	Correlation	Eastern Asia	SW and FW	10–20	10–20	-	10–20	-	-	AD (HNO₃ + H_2_O_2_)	-	LM	Valves
Lunetta et al. (2013)	19	To investigate diatom content in non-drowned bodies and diatom penetration into cadavers	Correlation	Northern Europe	Diatom enriched water	19–30	23–33	-	23–31	24–36	3–21	AD (HNO₃ + H_2_O_2_)	Entire pellet	LM	Whole diatoms and fragments of frustules
Lin et al. (2014)	120	To comprehensively study the presence of diatoms in lung tissue and the sphenoid sinus fluid collected during an autopsy of suspected drowning	Correlation	Eastern Asia	SW, FW and TP	5	-	-	-	-	-	AD (HNO₃ + HCl)	Entire precipitate (10 µl/slide)	LM	-
Kakizaki and Yukawa (2015)	10	To examine and validate how dissolving lung tissue using only commercial reagents can accelerate and simplify diatom extraction from suspected drowning cases	Correlation	Eastern Asia	SW and FW	AD = 5; ED = 2	10	-	10	-	-	AD (HNO₃ + H_2_O_2_) and ED† (Proteinase K + HCl)	All residue (200 µl/slide)	LM	Valves
Fucci et al. (2015)	20	To explore the possibility of adopting the EN 13946:2003 method for extraction of diatoms from organs in forensic practice by comparing it with a digestive method commonly used	Correlation	Southern Europe	SW and FW	10	10	-	10	10	10	AD (HCl) and AD^EN^ (H_2_O_2_ + HCl)	Entire pellet (300 µl/slide)	LM	Whole diatoms
Zhao et al. (2016)	64	To compare diatom numbers in lung tissue with drowning medium samples using a MD-VF-Auto SEM method	Correlation	Eastern Asia	-	2	-	-	-	-	-	MD (HNO₃ + H_2_O_2_)	-	Auto-SEM	Whole diatoms
Zhao et al. (2017)	128	To demonstrate the specificity and sensitivity of MD-VF-Auto SEM and the value of quantitative analysis of diatoms in the diagnosis of drowning	Correlation	Eastern Asia	FW, BW and TW	2	10	-	10	-	-	MD (HNO₃ + H_2_O_2_)	-	Auto-SEM	Valves
Shen et al. (2019)	64	To identify differences in diatom quantities between false-positive and true drowning	Correlation	Eastern Asia	-	10	10	-	10	-	-	MD (HNO₃ + H_2_O_2_)	-	Auto-SEM	Valves
Kihara et al. (2021)	44	To test the hypothesis that diatoms accumulate in the lungs through respiratory movements and to identify L/W ratios thresholds of actual drowning and postmortem immersion cases	Correlation	Eastern Asia	FW	< 25	-	-	-	-	-	AD (HNO₃)	Half of the precipitate (100 µL/slide)	LM	Valves
Kakizaki et al. (2022)	20	To Investigate the persistence of diatoms in Kjeldahl flasks used for testing	Causal-effect	Eastern Asia	SW and FW	2	10	-	10	-	-	AD (HNO₃ + H_2_O_2_)	All residue (100 µL/slide)	LM	Complete diatom frustule; connected frustules; destroyed valves (over half retained); small diatoms (5–7 μm) excluded
Sonoda et al. (2022)	78	To determine if reuse of Kjeldahl flasks causes false-positive diatom results and evaluate diatom presence in organs and blood as reliable evidence for drowning, using only new flasks to avoid contamination	Causal-effect	Eastern Asia	SW, FW, BW and TP	2	10	-	10	-	-	AD (HNO₃ + H_2_O_2_)	All residue (100 µL/slide)	LM	Complete diatom frustule; connected frustules; destroyed valves (over half retained)
Hagen et al. (2023)	5	To determine whether a modified MD-VF-SEM method can provide more reliable evidence of drowning compared to traditional methods	Causal-effect	Continental Europe	FW	10	-	-	-	-	-	Modified MD	-	SEM	-

SW = saltwater. FW = fresh water. TP = tap water. BW = brackish water. LM = Light microscopy. SEM = Scanning electron microscopy. AD = acid digestion *Weight of sample assumed to be weight of digested and analyzed sample unless specified. ED = enzymatic digestion. †= Rapid enzymatic digestion (proteinase K + buffer ATL, 5 N HCl). MD = microwave digestion. - = Not done/determined/comparable. ^EN^= digestion with 40% H_2_O_2_ + 1 M HCl according to EN13946

The tissue size sampled in each individual case ranged among these studies between 2 and 33 g for lung and 2 and 36 g for any closed organ (Table 4). Whereas lung tissue was analyzed in all the 17 studies, variance was greater among the closed organs, with the closed organs studied being brain, liver, spleen, kidney, and bone marrow. Five studies investigated 3 to 5 of these closed organs, another five studies 2 closed organs, and two studies a single closed organ, whereas five studies had no internal organs analyzed. Overall, 11 studies examined liver and renal tissue, four, bone marrow, and another four, brain tissue. Spleen tissue was considered in only one single study. It should also be noted that in studies with both drowning and non-drowning controls, tissue sampling was not matched between drowning and control groups, though lung tissue was always investigated (Table 4).

Since the siliceous diatom exoskeleton is highly resistant to biological decomposition, all studies used either acid- or enzymatic-based procedures to extract diatoms from tissues. Ten studies (58%) used acid-digestion methods, three (18%) enzymatic digestion, and four (24%) microwave digestion in association with HNO_3_ + H_2_O_2_ (Table 4). The acid extraction predominately consisted of a combination of HNO_3_ and H_2_O_2_ (four studies), although variations using H_2_SO_4_ + H_2_O_2_ (two studies), HCl + H_2_O_2_ (one study) and HNO_3_ + HCl (one study examining the EN13946 protocol) were also used. Two studies used only a single acid that being either HNO_3_ or HCl. The three studies (18%) based on enzymatic digestion, all used proteinase K, though one also in combination with HCl and another with H_2_O_2_. Concerning the analysis of digested tissues for diatoms, light microscopy was used in 13 (76%) among the 17 studies, whereas in the remaining four (24%) electron microscopy (SEM) served for identification, counting, and taxonomic classification of diatoms.

Further analysis of laboratory procedures demonstrated that of the 13 studies analyzing diatoms using light microscopy, only nine mentioned that “all sediment/residue” or “entire pellet/precipitate” was transferred onto the microscope slides, whereas in one the supernatant was analyzed (Table 4). No study mentioned the number of microscope slides used for each digested tissue nor mentioned whether only an aliquot of diatoms was counted and the total concentration for each organ was then extrapolated based on a partial count. With reference to microscopic findings, 8 studies (50%) reported quantities as whole diatoms and fragmented frustules were also counted if larger than half a diatom. Five studies (32%) reported findings as valves, and another three studies (19%) did not disclose their reporting criteria.

### Diatom concentrations in individual studies

Table 5 summarizes the average diatom concentrations recorded in each selected study, extrapolated to diatoms / 10 g where possible. Table S1 summarizes the range of diatom concentrations reported in each study, extrapolated to diatoms / 10 g where possible (Supplementary). Regardless of drowning media and of sampling and laboratory procedures, all studies revealed diatoms in lung tissues of individuals for whom the COD was drowning. The lowest concentrations of diatoms were observable in bathtub drownings (3.88 diatoms / 10 g), where the tap water contained 0.4 diatoms / ml. Freshwater drownings showed a range between 40 and 411,354 diatoms / 10 g for the lung, and lung tissue was noted to contain greater concentrations of diatoms than saltwater (range = 33.5 to 7162 diatoms / 10 g) and brackish water (average = 106,714 diatoms / 10 g based on one single study), though this was not the case for closed organs. Overall, drowning victims exhibited diatom / 10 g (tissue) ranges that ranged from 0.1 to 36.7 in liver, 0.1 to 38.3 in kidney, 2.5 to 160 in brain, and 7.4 to 28 in bone marrow.

**Table 5:** Comparison of diatom quan﻿tifications between studies averaged and extrapolated to number of diatoms/ 10 g of tissue

			Drowning							Non-drowning (land)							Non-drowning (immersed)						
References	Diatom/ml	Isolation method	Subjects	Lungs	Liver	Spleen	Kidney	Brain	Bone marrow	Subjects	Lungs	Liver	Spleen	Kidney	Brain	Bone marrow	Subjects	Lungs	Liver	Spleen	Kidney	Brain	Bone marrow
Ludes et al. (1994)	162	AD	*n* = 12	69.8	2.5	-	2.1	2.5	-	*n* = 5	0.0	0.0	-	0.0	0.0	-	-						
		ED		71.7	2.3	-	3.7	2.8	-														
Krstic et al. (2002)	-	AD	*n* = 22	197 (*n* = 19)	33.2 (*n* = 19)	-	37.6 (*n* = 21)	160 (*n* = 18)	28.0 (*n* = 15)	*n* = 1	407	64.5	-	144	204	110	-						
Takeichi and Kitamura (2009)	-	ED^3^	*n =* 6	595	-	-	-	-	-	-							*-*						
Ago et al. (2011)	0.4	AD	*n* = 9	3.88	0.19	0.19	0.08	-	-	-							*n* = 1	1.1	0.0	0.0	0.9	-	-
	2.1 ^IC^																						
Bartolotti et al. (2011)	-	AD	*n* = 20	348.2	-	-	-	-	7.4	*n* = 45	0.0	-	-	-	-	0.0	-						
Kakizaki et al. (2011)	^SW^ 23.8	AD	*n* = 11	2821	0.1	-	0.1	-	-	*n* = 1	0.0	0.0	-	0.0	-	-	-	-	-	-	-	-	-
	^FW^ 2756		*n* = 8	114796	0.4		1.6										*n* = 2	105^1^	20^1^		15^1^		
Lunetta et al. (2013)	77610	AD	-							*n* = 14	0.1	0.0	-	0.0	0.0	0.4	*n* = 5*	> 180	0.0	0.0	-	0.0	0.0
Lin et al. (2014)	-	AD	*n* = 84	^SW^ 185 (*n* = 37)	-	-	-	-	-	*n* = 20	0.0	-	-	-	-	-	*n* = 6	5230 (*n* = 2)	-	-	-	-	-
				^FW^ 2434 (*n* = 47)																			
Kakizaki and Yukawa (2015)	389	ED†	*n* = 10	14570	-	-	-	-	-	-							-						
		AD		5995	17.5		32.5																
Fucci et al. (2015)	-	AD	^SW^*n* = 7	33.5	26.7	-	22.4	32.4	10.3	-							-						
			^FW^*n =* 3	40.0	25.0		23.6	34.0	16.6														
		AD^EN^	^SW^*n* = 7	49.3	30.4		38.3	50.7	16.7														
			^FW^*n =* 3	63.3	36.7		33.0	43.3	26.0														
Zhao et al. (2016)	1207	MD-VF-SEM	*n* = 56	524937	-	-	-	-	-	-							*n* = 8	3021	-	-	-	-	-
	138^IC^																						
Zhao et al. (2017)	977	MD-VF-SEM	*n* = 115	67729	20.3	-	20.5	-	-	-							*n* = 13	532	0.0	-	0.0	-	-
	269 ^IC^																						
Shen et al. (2019)	-	MD-VF-SEM	*n* = 32	59324	36.6	-	25.5	-	-	*n* = 32	2.1	2.6	-	2.2	-	-	-						
Kihara et al. (2021)	1845	AD	*n* = 40	18571	-	-	-	-	-	-							*n* = 4	23.7	-	-	-	-	-
	5698^IC^																						
Kakizaki et al. (2022)	8399	AD	*n =* 20	20421	0.3 *(n* = 12)	-	0.3	-	-	-							-						
Sonoda et al. (2022)	^SW^ 80.3	AD	*n =* 27	7162	0.1	-	0.4	-	-	*n =* 4	0.0	0.0	-	0.3	-	-	*n =* 8	3.6^2^	1.0^2^	-	0.6^2^	-	-
	^FW^ 2534		*n =* 22	411354	0.1		18.3																
	^BW^ 301		*n =* 7	106714	0.1		0.4																
	^TW^ 0		*n =* 10	2.0	0.1		0.2																
Hagen et al. (2023)	57298	MD-VF-SEM	*n =* 5	49704	-	-	-	-	-	-							-						

*AD* = acid digestion using concentrated inorganic (nitric/sulphuric/acetic/hydrochloric/hydrogen peroxide) acid. *EZ* = enzymatic digestion using proteinase K. †= Rapid enzymatic digestion (proteinase K + buffer ATL, 5 N hydrochloric acid). *MD-VF-SEM* = microwave digested vacuum filtration automated SEM purification method. *SW* = Saltwater, *FW* = Freshwater, *BW* = Brackish-water, *TW* = tap water. - = Not determined/not comparable. *Postmortem diatom-infused lungs in situ. ^IC^ 2.1 specific to non-drowning immersed control. ^1^Medium had an average of 17 400 diatoms/ml. ^2^ Water type not specified, total average 1876 diatoms/ml. ^EN^= digestion with 40% hydrogen peroxide plus 1 M hydrochloric acid (EN13946:2003). ^3^ unfixed tissue

In terms of digestion methods, results from two studies that compared acid with enzymatic techniques showed marginally greater average concentrations of diatoms in lung tissue following enzyme digestion than acid digestion (Table 5). Data for closed organs is scarce, as only a single study compared both methods, and that study indicated a similar marginally greater average following enzyme digestion. Diatom concentrations observed in studies employing MD-VF-SEM were overall much greater than both acid and enzyme-based methods, though they exhibited a smaller range.

Diatoms were also identified in both non-drowning control groups, in four studies of the non-drowned, found on dry land group, and in eight studies investigating non-drowned but immersed. In the non-drowned (dry land) group, average diatom concentrations in lung tissue ranged between 0 and 407 diatoms / 10 g. The higher value is, however, based on only a single study, examining only a single individual. Further analysis reveals that of the eight studies that investigated lung tissue in non-drowned individuals found on land, four did not identify any diatoms. For closed organs, diatoms were identified in liver tissue in two studies, exhibiting a range of 2.6 to 64.5 diatoms / 10 g. Three studies identified diatoms in kidney tissue with a range of 0.3 to 144 diatoms/ 10 g, and two studies examining bone marrow demonstrated a range of 0.4 to 110 diatoms / 10 g. As to the non-drowned control group characterized by PM immersion/submersion, all eight studies revealed diatoms in the lung tissues, with a range of 1.1 to 5230 diatoms / 10 g of lung tissue. No diatoms were identified in bone marrow, spleen or brain, though two studies examining liver tissue noted diatom concentrations between 1 and 20 diatoms / 10 g, and three studies examining kidney tissue documented diatom concentrations of 0.6 to 15 / 10 g (Table 5).

## Discussion

Despite the advancement of scientific methodological rigor and the growing emphasis on evidence-based practice in forensic medicine, the evidentiary value of the diatom test for the PM diagnosis of drowning remains difficult to assess. An increasing number of studies have recently sought to refine analysis methods [20–27] and to address quantitative and qualitative aspects [20, 28–31], as well as the issue of false-positive diatom findings in non-drowned controls [27, 32]. While some reviews have summarized the existing literature on the diatom test [5, 7, 18, 33–35], no systematic review on the topic exists.

In contrast to typical literature reviews that address a general topic or problem of interest, systematic reviews deal with clear research questions that can be answered by transparent methods enabling informed decision-making based on the best available evidence [36–38]. We therefore conducted a systematic review with the aim to examine “how the presence of PM diatoms may be interpreted in medico-legal investigations of fatal drowning”.

Results from the 17 selected studies (from the initial 372 records identified), demonstrate that diatom concentrations are typically greater in the lungs and internal organs of drowning victims than in non-drowned control groups, that are either found on land or immersed PM. Such results, when considered in isolation, suggest that the diatom test could serve as effective supportive evidence for diagnosing drowning. However, considerable inconsistencies exist in the concentrations of diatoms detected in the lungs and internal organs of definite drowning victims (Table 5). The diagnostic value of diatoms in lung tissue alone is also controversial, as data from control groups in the selected studies suggests that during PM immersion, diatoms can indeed penetrate the airways passively.

Variations in study design, sample population, laboratory procedures and methodologies for analyzing diatom samples hinder the comparisons of results among the selected studies; they also hamper establishment of any cut-off concentration value to enable a drowning diagnosis based on diatoms in lung and other organ tissues. Reported cut-off concentration values for diagnosing drowning in the literature differ, with some studies proposing thresholds of ≥ 20 diatoms / 10 g for lung tissue and 5 diatoms /10 g for other internal-organ tissues [20, 27, 39–43]. Another proposal is ≥ 20 diatoms /100 g for lung tissue and 13 diatoms for liver tissue [44]. However, control cases have also been described as overlapping these cut-off values, exhibiting between 5 and 25 diatoms /100 g in lung tissue and up to 10 diatoms in closed organs [45]. Another proposal is that a threshold of 10 diatoms / 10 g of lung tissue is sufficient to indicate drowning when using the MD-VF-Auto SEM method [27]. Problematically, the overshadowing restriction that limits identifying an accurate cut-off value is the misleading assumption that, during the drowning process, each drowning victim inhales the same volume of liquid.

Ultimately, the same factors that hamper the establishment of cut-off concentrations of diatoms in drowning, also stem from issues relating to false-negative and -positive cases. These issues include lack of diatoms in drowning cases and discovery of diatoms in PM tissues of corpses with a COD other than drowning. These are two of the main criticisms levelled against the diatom test. Although the selected studies do report false-negative and positive results, they collectively fall short in thoroughly assessing such results’ impact on the evidentiary value of the test.

A common interpretation following diatom testing is that the absence of, or low concentrations of diatoms identified in PM tissue samples may be attributed to similarly low concentrations of diatoms in the drowning media or even to their absence [32, 46]. However, this interpretation overlooks a number of alternative factors that may also result in false-negative results. First, only a small volume of liquid is necessary to penetrate the airways and cause death by drowning [47], and cardiorespiratory arrest may occur during a very early stage of the drowning process. Secondly, inadequate tissue sampling during autopsy, including insufficient volume and number of samples, can result in low diatom concentrations [28]. A similar outcome will occur from the (partial) loss of diatoms during laboratory procedures such as tissue digestion, centrifugation, and transfer of pellets to microscope slides. Whatever the mechanism, a low concentration of diatoms in PM tissue will reduce the taxonomic spectrum available for comparison with the diatom species in the putative drowning medium.

In regard to false-positive results, a range of AM and PM contamination may account for detection of diatoms in non-drowned cases (and for an overestimation of diatoms in actual drowning cases). Detection of diatoms either in lung or in closed-organ tissues of submerged corpses that did not die from drowning may be interpreted as PM contamination [20, 23], thus limiting the diatom test’s reliability for a diagnosis of drowning [28]. Direct exposure to diatom-rich water (bi-cellular cultures of *Thalassiosira baltica* and *Thalassiosira levanderi*) via tracheostomy of the lungs of non-drowned cadavers with advanced putrefaction changes revealed, however, no diatoms during autopsy in closed organs 3–4 days after infusion [30]. Those authors demonstrated under controlled conditions, simulating extreme PM submersion, that the risk of false-positive results in closed organs due to PM diatom penetration in corpses is less significant than previously assumed [30]. On the other hand, passive PM penetration of liquid into the lungs should always be considered when interpreting diatom findings in the tissue. This is further supported by the fact that all selected studies indicate that all PM-immersed groups had diatoms in their lungs, albeit at lower concentrations than that identified in drowned victims. Interestingly, in a single study exposing whole cat lungs to diatom-enriched media *in vitro*, without any infusion, no diatom penetration of occurred [48].

PM contamination can also occur during autopsy, via the transfer of diatoms from the surface of the body to internal organs, between internal organs and during tissue sampling and laboratory procedures [31, 32, 41]. A prerequisite for diatom analysis is therefore the use of sterile or single-use instruments [28, 49]. As is frequent glove-changes and the use of bi- or tri-distilled H_2_O during centrifugation cycles and testing for other potential contamination sources, such as reagents [27].

Detection of diatoms unrelated to drowning can also result from AM diatom penetration. This can occur when victims are recovered from aquatic environments they were previously exposed to [50]. For instance, swimmers or divers can inhale diatom-rich liquid during earlier activities unrelated to the fatal drowning episode; or those living near the shore may inhale diatom species that are aerophilic [14, 27, 51]. Consumption of seafood such as shellfish, containing a high quantity of diatoms [52] may be an additional source of AM diatom penetration. This is, however, controversial, as little evidence demonstrates that diatom frustules can enter the systemic circulation via the human gastrointestinal tract, although simulated gastric lavage in anesthetized experimental animals has recently suggested otherwise [53].

To address these gaps, studies must employ stricter, and standardized protocols encompassing tissue sampling, laboratory procedures, diatom counting, and taxonomic analysis. A similar call for standardization was recently made by Ludes et al. [35], and our findings further support this view, as the selected studies only partially adhered to standardized methods. Future studies should aim at optimizing the quantitative and qualitative assessment of diatoms to establish *reference intervals* for the lung and internal organ tissues applicable to drowning and non-drowning cases. This will also reduce the ambiguity surrounding false-positive and false-negative results.

Although lung tissue was analyzed in all selected studies, differences emerged concerning the type and number of closed organs sampled. Surprisingly, only four studies examined bone marrow. The volume of samples varied between studies examining the same tissue, with lower volumes of lung tissue typically sampled than the volumes of closed organs [23, 24, 26, 31, 32, 41]. It could be argued that when establishing reference intervals for drowning, smaller samples may yield fewer diatoms. On the other hand, when sampling tissue such as the lungs, high concentrations of diatoms and other potential contaminants may interfere with diatom counting. This may also hinder taxonomic analysis making dilution of the sediment left after tissue digestion and centrifugation necessary. Furthermore, the sampling site in each organ should also be considered, due to the possible unequal distribution of diatoms. Importantly, having more than a single sample of adequate volume for each organ allows for the repetition of the diatom analysis to verify results. Standardizing sample collection is crucial for all diatom testing to ensure comparable quantitative results in both routine casework and control cases.

As to laboratory procedures, digestion of tissue samples, by means of chemical or enzymatic methods may lead to, at least partial loss of diatoms. Most of our selected studies (65%) employed chemical digestion (acids) due to its effectiveness in removing organic material and yielding diatoms with clean surfaces. However, the acid also causes a selective destruction of diatoms, especially of those with a weaker siliceous frustule [54, 55]. This can, in turn, result in underestimation of diatom concentration and a reduction in their taxonomic spectrum. The wide variability of chemical digestion protocols adopted in the selected studies reduces reliable comparisons of quantitative and qualitative results. In an effort to compare digestion methods, Fucci et al. [22] showed that the digestion protocol outlined in the European Standard for the routine sampling and preparation of benthic diatoms from rivers and lakes (EN13946), which utilizes 40% H_2_O_2_ and 1 M HCl, yielded higher diatom concentrations in both lung and closed-organ tissues, when compared to 37% HCl alone.

Compared to chemical methods, enzymatic digestion methods [20, 21, 23, 56] offer the advantage of being environmentally safer, albeit at a higher cost and with weaker tissue-digestion effectiveness [55]. This may in principle, limit the volume of tissues to be studied and may result in suboptimal cleaning of diatom surfaces. Poor cleaning can, in turn, pose challenges to taxonomic diatom identification. Although few studies (18%) included in this review have considered enzymatic methods, comparisons with acid digestion demonstrated similar results in lung, liver, and kidney tissues [20].

Analysis of freshwater diatoms (specifically within the *Cyclotella* and *Cybella* genera) spiked into 2 g of kidney, liver, and bone marrow tissue of rabbits suggests that proteinase K has a greater extracting effectiveness than either HNO_3_ + H_2_O_2_ or Soluene-350 digestion [55]. For seawater diatoms (within the *Navicula* genera), proteinase K again showed in the same comparative study greater reclaiming ratios [55]. Kjeldahl flasks, especially when re-used or damaged, is also a potential contributor to false-negative and -positive results [31]. The siliceous coating of the diatom frustule can adhere to the flask’s inner glass surface during the heating procedure, or alternatively, diatom fragments can be trapped in small defects of the flasks and subsequently be released when the flask is re-used [20, 30, 31].

The development of the Microwave Digestion - Vacuum Filtration - Automated Scanning Electron Microscopy (MD-VF-Auto SEM) method addresses the partial loss of diatoms during acid digestion and centrifugation [25, 27]. Moreover, by also using an automated SEM system to capture images, the method enables a more detailed and comprehensive quantitative and qualitative analysis of diatoms compared to analysis by traditional light microscopy [26, 57]. However, this approach is currently only employed at a limited number of institutions, and appears to yield considerably higher diatom quantities in the lungs of drowning victims when compared to either the acid or the enzymatic digestion protocols [24, 26, 27]. Zhao et al. [26], using the MD-VF-Auto SEM method, normalized diatom concentrations by calculating the lung-to-water-sample diatom concentration-rate ratio (L/D). Further studies are necessary to assess the practical utility of this ratio in distinguishing drowning from merely PM immersion and distinguishing COD other than drowning.

Once tissue digestion is performed, either by chemical or enzymatic methods, the digestion product must be decanted and transferred into dedicated tubes for centrifugation and washing cycles. Such cycles too, should be standardized since a selective loss of diatoms can in principle, occur during these steps [25, 49]. As highlighted in our results, none of the selected studies had included any positive control during tissue digestion and centrifugation (Table 3). To better understand the potential loss of diatoms during the tissue digestion and centrifugation steps, spiking samples during routine casework as a positive control, with a known monocellulate diatom species and concentration may help determine digestion and centrifugation effectiveness. Such a spiking-and-recovery test was conducted by Seo et al. [58] to evaluate the effectiveness of a DNA co-precipitation method for the detection of diatoms in heart blood. The spike, which was introduced prior to digestion using proteinase K in non-drowned control subjects, showed recovery rates ranging from 88.4 to 100%. By introducing a known concentration of a distinct diatom species to a tissue sample before laboratory processing, a recovery analysis can provide valuable insights into the robustness and effectiveness of laboratory procedures and eventually offer insights for better interpretation of casework with low diatom concentrations or false-negative cases.

As mentioned, quantitative studies that aim to establish cut-off values and address the issue of false-positive and -negative issues are further hampered by lack of standardization for the transfer of digested tissue aliquots onto microscope slides. The 17 selected studies only rarely mention whether the entire pellet is transferred after centrifugation and washing cycles into one or more microscope slides, nor do they mention whether the centrifugation tubes are washed to collect any residual diatoms that may remain on the bottom and on internal surfaces before being transferred.

Equally crucial are the criteria used for diatom counting, as the studies consider interchangeable terms such as whole diatoms, valves, or fragments of diatoms. Although the inclusion or exclusion of fragments are at times detailed in the studies, counting is vulnerable to subjectivity. Moreover, because only separate valves are detected under a microscope following tissue digestion, reporting findings as diatom frustules, is misleading.

Lastly, although taxonomic analysis of diatoms is frequently performed by experienced in-house diatom experts, this task may be inherently subjective, associated with an increased risk of bias, and a lack of consistency and transparency. To mitigate this, diatom analysis focusing on control series in non-drowned bodies and analysis addressing laboratory procedures, should be conducted by an independent taxonomist without ties to routine PM diatom analyses in drowning cases.

Automated systems using artificial intelligence (AI) are a promising tool to standardize the identification, count, taxonomic classification, and comparison of diatoms, which potentially could mitigate subjectivity and human error. Several studies hav explored the potential of machine learning to train automated diatom detection and have obtained promising results [43, 59–61]. Nevertheless, difficulties remain in training the AI to differentiate between diatoms and larger debris, as well to identify species-specific surface details of diatoms partially covered by undigested particles, and to discriminate among diatom species similar in size and shape [43]. Although further studies, along with cost assessments and personnel training, are necessary before implementation of automated diatom identification as standard practice, this approach has the potential to enhance workflow efficiency [62] and improve systematic taxonomic assessments [61].

Standardization and calibration are well-known practices among taxonomists involved in quantitative and qualitative analysis of diatoms in aquatic environments [63, 64]. For instance, in order to establish consistent and comparative river-monitoring data in Sweden, trained diatomists adhere to the European Standards for sampling, isolation, and classification [63, 65] and participate in intercalibration exercises organized by the Nordic–Baltic Network for Benthic Algae in Freshwater (NORBAF). Research demonstrates that harmonization of taxonomic identification is more important than mere experience for achieving higher levels of similarity and reproducibility of results [63]. Moreover, data provided by systematic monitoring efforts, including synchronized taxonomy lists, are shared through platforms such as the Arctic Biodiversity Data Service and updated via initiatives such as the Circumpolar Biodiversity Monitoring Programme (CBMP) [65]. Systematic monitoring of diatoms in well-defined aquatic settings and comparison with the taxonomic profile of diatoms detected PM in the victims’ tissues may also be helpful in forensic medicine for the assessment of the site and time of drowning [66, 67]. It may therefore be recommended that until validated automated systems are in place, identification of diatoms should be conducted according to standardized methods such as the EN14407 guidelines for identification, enumeration, and interpretation of benthic diatom samples from running waters [68]. Additionally, participation of forensic laboratories in intercalibration exercises is highly advisable to further enhance accuracy and consistency in laboratory procedures and taxonomic assessments.

## Limitations of study

This review faces a number of limitations originating from the predetermined constraints dictated by the strict nature of how research questions are formulated in systematic reviews [69]. There exists, therefore, a possible selection bias, with only 17 studies deemed eligible. This should be considered when interpreting the review’s outcomes. For instance, the extrapolation of original diatom count in tissues into concentration (number of diatoms / 10 g tissue), required specific information (volume of tissue sampled at autopsy and digested, aliquot transferred onto the microscope glass, reporting of precise diatom quantities identified) that were not always available. The lack of such information has led to the exclusion of several otherwise potentially valuable studies.

This systematic review was limited to human studies published in English peer-reviewed journals. Several studies in other languages (such as German, French, and Italian), studies based on animal experiments, as well as data published in monographs and doctoral thesis on the diatom method for the diagnosis of drowning were therefore not considered here.

Lastly, data available in institutions that routinely utilize the diatom method for the diagnosis of drowning, but which were not developed into original contributions published in peer-reviewed journals, were also not taken into consideration here. Therefore, this systematic review may overlook the experience of researchers within the field.

## Conclusions

In this systematic review, we have investigated how the presence of diatoms PM may be interpreted in medico-legal investigations of drowning by examining reported diatom concentrations in suspected drownings, non-drowned land-based controls, and non-drowned immersed controls. Results, considered in isolation, indicate that drowning victims exhibit greater concentrations of diatoms in their lungs and closed organs when compared to concentrations in either a land- or immersion-based control group. However, findings also show clearly that current quantitative research is insufficiently comparable and is of limited value in establishing reliable cut-off values for drowning. Additionally, these studies fall short in addressing the main criticisms levelled against the diatom method, namely the issues of false-positive and false-negative cases. Further well-designed studies are necessary to harmonize methods and to implement standardized procedures in routine casework. This will overcome the crucial obstacles to accepting the evidentiary value of the diatom test in the courtroom.

## Supplementary information

Below is the link to the supplementary material.

ESM 1 (DOCX 25.8 KB)

## Data Availability

Not applicable. Data is based on published studies.
