# Supplementary material for: The medico-legal interpretation of diatom findings for the diagnosis of fatal drowning: a systematic review
Source: Int J Legal Med. 2025 Jan 14;139(2):729–46. doi: 10.1007/s00414-024-03397-8 (PMC11850479; doi:10.1007/s00414-024-03397-8)
Supplement: Supplementary file 1 — (DOCX 25.8 KB) [file 414_2024_3397_MOESM1_ESM.docx]

**Table S1. Comparison of diatom quantifications between studies, ranges (min-max) extrapolated to number of diatoms/ 10 g of tissue**

|  | | | **Drowning** | | | | | | | **Non-drowning (land)** | | | | | | | **Non-drowning (immersed)** | | | | | | |
| --- | --- | --- | --- | --- | --- | --- | --- | --- | --- | --- | --- | --- | --- | --- | --- | --- | --- | --- | --- | --- | --- | --- | --- |
| **References** | **Diatom/ml** | **Isolation method** | **Subjects** | **Lungs** | **Liver** | **Spleen** | **Kidney** | **Brain** | **Bone marrow** | **Subjects** | **Lungs** | **Liver** | **Spleen** | **Kidney** | **Brain** | **Bone marrow** | **Subjects** | **Lungs** | **Liver** | **Spleen** | **Kidney** | **Brain** | **Bone marrow** |
| Ludes et al (1994) | 150-410 | AD | *n*=12 | 4-108 | 0-10 | - | 0-15 | 0-10 | - | *n*=5 | NA | NA | - | NA | NA | - | - | | | | | | |
|  |  | ED |  | 0-105 | 0-10 | - | 0-10 | 0-11 | - |  |  |  |  |  |  |  |  |  |  |  |  |  |  |
| Krstic et al (2002) | - | AD | *n*=22 | 0-1330 (*n*=19) | 0-185  (*n*=19) | - | 0-310 (*n*=21) | 0-980  (*n*=18) | 0-285 (*n*=15) | *n*=1 | NA | NA | - | NA | NA | NA | - | | | | | | |
| Takeichi and Kitamura (2009) | - | ED^3^ | *n=*6 | 25–1533 | - | - | - | - | - | - | | | | | | | *-* | | | | | | |
| Ago et al (2011) | 0-1.8 | AD | *n*=9 | 0–28 | 0–1.1 | 0–1.3 | 0–0.9 | - | - | - | | | | | | | *n*=1 | NA | NA | NA | NA | - | - |
|  | NA ^IC^ |  |  |  |  |  |  |  |  |  |  |  |  |  |  |  |  |  |  |  |  |  |  |
| Bartolotti et al (2011) | - | AD | *n*=20 | 25–1000 | - | - | - | - | 0–800 | *n*=45 | NA | - | - | - | - | NA | - | | | | | | |
| Kakizaki et al (2011) | ^SW^ 2–50 | AD | *n*=11 | 670–52000 | 0–10 | - | 0–40 | - | - | *n*=1 | NA | NA | - | NA | - | - | - | - | - | - | - | - | - |
|  | ^FW^ 33–13000 |  | *n*=8 | 380–2000000 | 0–50 |  | 0–300 |  |  |  |  |  |  |  |  |  | *n*=2 | 0–210^1^ | NA |  | 0–30^1^ |  |  |
| Lunetta et al (2013) | NA | AD | - | | | | | | | *n*=14 | 0–4 | NA | - | NA | NA | 0–12 | *n*=5* | >180 | NA | NA | - | NA | NA |
| Lin et al (2014) | - | AD | *n*=94 | ^SW^ NA | - | - | - | - | - | *n*=20 | NA | - | - | - | - | - | *n*=6 | NA | - | - | - | - | - |
|  |  |  |  | ^FW^ NA |  |  |  |  |  |  |  |  |  |  |  |  |  |  |  |  |  |  |  |
| Kakizaki and Yukawa (2015) | 13–900 | ED† | *n*=10 | 600–230000 | - | - | - | - | - | - | | | | | | | - | | | | | | |
|  |  | AD |  | 480–73000 | 10–40 |  | 10–90 |  |  |  |  |  |  |  |  |  |  |  |  |  |  |  |  |
| Fucci et al (2015) | - | AD | ^SW^ *n*=7 | 20–60 | 10–60 | - | 10–40 | 32.4 | 5–15 | - | | | | | | | - | | | | | | |
|  |  |  | ^FW^ *n=*3 | 30–55 | 25–30 |  | 20–30 | 22–50 | 10–20 |  |  |  |  |  |  |  |  |  |  |  |  |  |  |
|  |  | AD^EN^ | ^SW^ *n*=7 | 35–80 | 20–45 |  | 25–62 | 20–80 | 10–22 |  |  |  |  |  |  |  |  |  |  |  |  |  |  |
|  |  |  | ^FW^ *n=*3 | 40–80 | 20–60 |  | 24–50 | 30–60 | 15–30 |  |  |  |  |  |  |  |  |  |  |  |  |  |  |
| Zhao et al (2016) | 3.6–9500 | MD-VF-SEM | *n*=56 | 260–7577425 | - | - | - | - | - | - | | | | | | | *n*=8 | 75–9450 | - | - | - | - | - |
|  | 2.1-537^IC^ |  |  |  |  |  |  |  |  |  |  |  |  |  |  |  |  |  |  |  |  |  |  |
| Zhao et al (2017) | NA | MD-VF-SEM | *n*=115 | NA | NA | - | NA | - | - | - | | | | | | | *n*=13 | 15–1890 | NA | - | NA | - | - |
|  | 0.4–616 ^IC^ |  |  |  |  |  |  |  |  |  |  |  |  |  |  |  |  |  |  |  |  |  |  |
| Shen et al (2019) | - | MD-VF-SEM | *n*=32 | 187–699218 | 2–426 | - | 3–184 | - | - | *n*=32 | 0–35 | 0–20 | - | 0–9 | - | - | - | | | | | | |
| Kihara et al (2021) | 2–946 | AD | *n*=40 | 60–223200 | - | - | - | - | - | - | | | | | | | *n*=4 | 1–71 | - | - | - | - | - |
|  | 224-1488^IC^ |  |  |  |  |  |  |  |  |  |  |  |  |  |  |  |  |  |  |  |  |  |  |
| Kakizaki et al (2022) | 64-100000 | AD | *n=*20 | 50-315000 | 0-20 *(n*=12) | - | 0-20 | - | - | - | | | | | | | - | | | | | | |
| Sonoda et al (2022) | ^SW^ 2-1000 | AD | *n=*27 | 10-90000 | 0-1 | - | 0-1 | - | - | *n=*4 | NA | NA | - | 0-1 | - | - | *n=*8 | 0-40^2^ | 0-5^2^ | - | 0-2^2^ | - | - |
|  | ^FW^ 23-16000 |  | *n=*22 | 300-8500000 | 0-0.5 |  | 0-4000 |  |  |  |  |  |  |  |  |  |  |  |  |  |  |  |  |
|  | ^BW^ 90-1200 |  | *n=*7 | 2300-49000 | 0-1 |  | 0-3 |  |  |  |  |  |  |  |  |  |  |  |  |  |  |  |  |
|  | ^TW^ NA |  | *n=*10 | 0-20 | 0-1 |  | 0-2 |  |  |  |  |  |  |  |  |  |  |  |  |  |  |  |  |
| Hagen et al (2023) | 315-24148 | MD-VF-SEM | *n=*5 | 24100-119280 | - | - | - | - | - | - | | | | | | | - | | | | | | |

NA= not applicable/ not attainable. AD= acid digestion using concentrated inorganic (nitric/sulphuric/acetic/hydrochloric/hydrogen peroxide) acid. EZ = enzymatic digestion using proteinase K. †= Rapid enzymatic digestion (proteinase K + buffer ATL, 5N hydrochloric acid). MD-VF-SEM = microwave digested vacuum filtration automated SEM purification method. SW= Saltwater, FW = Freshwater, BW= Brackish-water, TW = tap water. - = Not determined/not comparable. *Postmortem diatom-infused lungs *in situ*. ^IC^ 2.1 specific to non-drowning immersed control. ^1^Medium range 1400-32000 diatoms/ml. ^2^ Water type not specified, total average 1876 diatoms/ml. ^EN^= digestion with 40% hydrogen peroxide plus 1M hydrochloric acid (EN13946:2003). ^3^ unfixed tissue.
